# Exploring Spatial Trends and Influencing Factors for Gastric Cancer Based on Bayesian Statistics: A Case Study of Shanxi, China

**DOI:** 10.3390/ijerph15091824

**Published:** 2018-08-23

**Authors:** Gehong Zhang, Junming Li, Sijin Li, Yang Wang

**Affiliations:** 1 Medical Imaging Department, Shanxi Medical University, Taiyuan 030001, Shanxi, China; zhanggh88666@sina.com (G.Z.); wy826618101@163.com (Y.W.); 2 School of Statistics, Shanxi University of Finance and Economics, Taiyuan 030006, Shanxi, China; Lijm@sxufe.edu.cn

**Keywords:** gastric cancer, spatial variability, influencing factors, dietary structure

## Abstract

Gastric cancer (GC) is the fourth most common type of cancer and the second leading cause of cancer-related deaths worldwide. To detect the spatial trends of GC risk based on hospital-diagnosed patients, this study presented a selection probability model and integrated it into the Bayesian spatial statistical model. Then, the spatial pattern of GC risk in Shanxi Province in north central China was estimated. In addition, factors influencing GC were investigated mainly using the Bayesian Lasso model. The spatial variability of GC risk in Shanxi has the conspicuous feature of being ‘high in the south and low in the north’. The highest GC relative risk was 1.291 (95% highest posterior density: 0.789–4.002). The univariable analysis and Bayesian Lasso regression results showed that a diverse dietary structure and increased consumption of beef and cow milk were significantly (*p* ≤ 0.08) and in high probability (greater than 68%) negatively associated with GC risk. Pork production per capita has a positive correlation with GC risk. Moreover, four geographic factors, namely, temperature, terrain, vegetation cover, and precipitation, showed significant (*p* < 0.05) associations with GC risk based on univariable analysis, and associated with GC risks in high probability (greater than 60%) inferred from Bayesian Lasso regression model.

## 1. Introduction

Gastric cancer (GC), or stomach cancer, is a serious health problem. It is the fourth most common type of cancer and the second leading cause of cancer-related deaths worldwide [1]. More than 950,000 new cases are diagnosed annually [1]. According to estimates, approximately 720,000 patients died from stomach cancer in 2012 [2]. In particular, East Asia accounts for more than half of GC cases globally [3]. Furthermore, 679,100 new cases of GC are estimated to be diagnosed in China annually [4]. In addition, approximately half of the world’s GC cases occur in China [5]. The 5-year survival rate for stomach cancer is low because more than 80% of Chinese patients are diagnosed as advanced stage [6]. An estimated 498,000 Chinese people died from GC in 2015 [4]. GC is a major contributor to the global burden of disability-adjusted life-years due to cancer in men [7]. The burden of GC is very high in Asia [8], particularly in China. The average expenditure per patient with GC is $9891 ($9606–$10,176), which is surpassed only by colorectal, oesophageal, and lung cancers; moreover, this expenditure is nearly 1.15 times the annual household income [9]. In addition, expenditures increase from stage I to stage IV GC [9]. Despite the nationwide conditions of a developing country with a large population, the Chinese national total health care bill as a proportion of gross domestic product (GDP) is less than that in most countries. Given the shortage of government investment, the high financial burden of stomach cancer is ultimately transferred to a patient’s family, society and medical institutions. Nevertheless, some studies suggest that the early detection and treatment of GC is the primary way to reduce the disease burden and mortality [10,11,12,13].

Many studies have investigated the risk factors for GC. Like other cancers, GC is a multifactorial disease, and genetic and environmental factors play a role in its pathology [13]. However, except for gender and age, other factors such as alcohol consumption, smoking, and *Helicobacter pylori* (*H. pylori*) infections are potential risk factors that must be continuously studied from diverse perspectives [13]. The incidence of GC increases significantly with age [14,15]. Males have a higher risk than females, although the reasons for this difference are not clear [16]. Tobacco smoking and alcohol consumption can increase the risk of GC [17,18,19,20]. A systematic review [21] of epidemiological data in Japan showed that the relative risk of GC in current smokers was 1.56 (95% CI 1.36–1.80) compared with that of never smokers. According to a Korean population-based cohort study, the odds ratio for developing GC for those who consumed more than 15 g of alcohol a day was 1.2 (95% CI 1.0–1.3) times that of non-drinkers [22]. Several studies have found that *H. pylori* is the most likely cause of GC [23], with an approximately 6-fold higher relative risk of noncardia GC [24]. Certain *H. pylori* types, particularly those positive for the virulence factor cytotoxin-associated gene A (CagA), are more likely a cause of GC [25,26,27]. *H. pylori* is a major risk factor for noncardia GC but not cardia GC in Western countries [24]. Nonetheless, several interventional studies from Asia [28,29,30,31] have shown that the eradication of *H. pylori* does not prevent the development of GC. Additional studies are necessary to address this issue. Dietary structure also plays an important role in GC. The World Cancer Research Fund/American Institute for Cancer Research (WCRF/AICR) has declared that “Salt, and salt-preserved foods, are probable causes of GC” [32]. A Japanese prospective study [33] and a Chinese provincial case-control study [34] found that vegetable and fruit intake was connected with a low risk of GC, likely due to antioxidant effects. High GC risks are associated with excessive dietary salt intake [35]. Additionally, smoked food was also a risk factor for GC [36,37]. In addition, several studies have reported that diets with high antioxidant levels [38] and diets with a high fibre content [39] are related to a lower GC risk; however, additional studies must be conducted [13]. In addition, other potential demographic risk factors for GC, e.g., obesity, physical activity, and oral hygiene, have also been investigated, yet the corresponding results remain unclear. Several studies have investigated the association between socio-economic factors and GC [40,41,42,43]. To our knowledge, little research about the geographic environmental risk factors for GC have been conducted. Yamaoka et al. [44] demonstrated that geographic differences in GC incidence can be explained by differences between *H. pylori* strains. 

In addition to risk factors, spatial trends of disease are also very important, particularly for developing prevention and control policies. In general, disease mapping requires a large amount of disease survey data, which involves significant labour and material resources. Moreover, some disease surveys cannot be conducted because of logistical restrictions. Conversely, hospital diagnostic case data are conveniently obtained and contain more potential information. However, hospital-diagnosed case data may contain bias and have a relatively small sample size. These limitations restrain mining hospital-diagnosed data to a certain extent. 

In view of these considerations, this study has four main goals: (a) to present a selection probability model that attempts to correct the bias in hospital diagnostic case data; (b) to explore the relevant factors of GC based on estimated spatial variability employing epidemiological methods, univariable analyses and multivariable regression models; (c) to overcome the problem of a small sample size by utilizing a Bayesian statistical framework integrated with the abovementioned models; and (d) to apply these methods to explore the spatial variation and influencing factors of GC in Shanxi in north central China, which has the highest GC incidence in China [34]. 

## 2. Materials and Methodologies

### 2.1. Study Materials

#### 2.1.1. Diagnosed Patients

Between April 2014 and June 2016, data on 379 diagnosed and surviving GC patients were collected from the First Hospital of Shanxi Medical University (FHSMU) in Taiyuan, Shanxi Province. 

**Figure 1:**
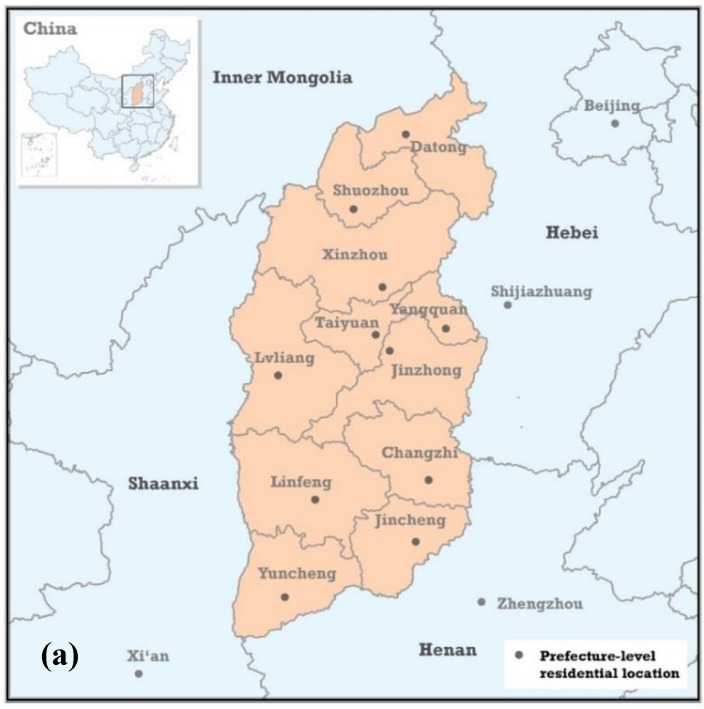

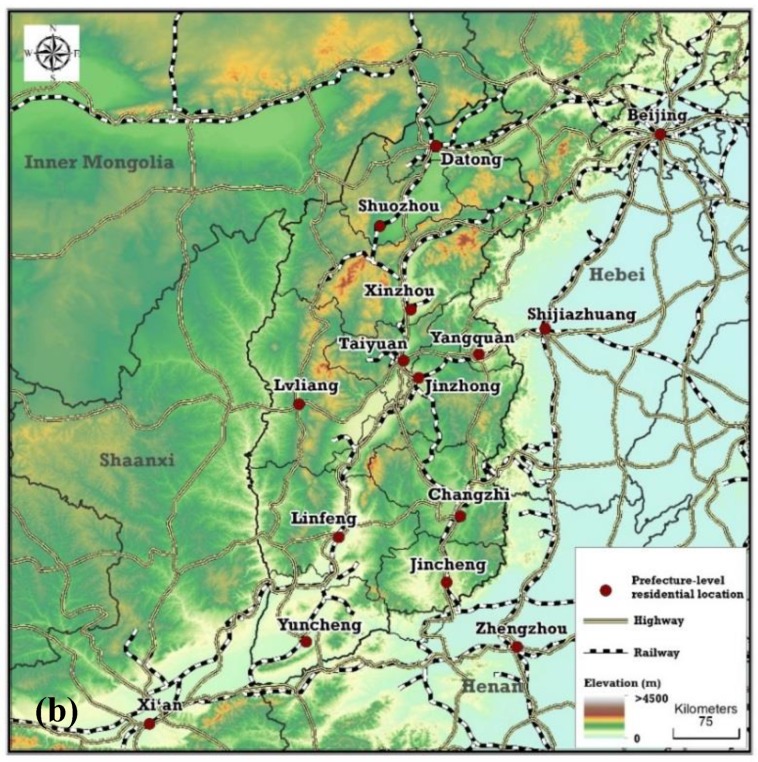
Location of Shanxi Province in China and the 11 prefecture-level administrative subdivisions of Shanxi Province (**a**); terrain and traffic network of highways and railways over Shanxi and its environs (**b**).

Of these 379 patients, 358 resided in 11 prefecture-level cities of Shanxi Province, e.g., Taiyuan, Datong, and Xinzhou, and 21 were from neighbouring provinces, e.g., Hebei, Henan, and Shaanxi. Shanxi Province is located in north central China and has four neighbouring provinces (Figure 1). It has a population of approximately 30 million. To ensure the integrity of the study region, the 358 patients residing in Shanxi were included (281 males and 77 females; average age 63 ± 12 years). Of these 358 patients, 346 were diagnosed with gastric adenocarcinomas (GACs), five with GC with signet ring cell carcinomas, and the remaining seven with an undetermined type of GC. These GC diagnoses were histologically confirmed by professional clinical doctors at the FHSMU. Most TNM stages were III and above; only 10 patients were diagnosed as TNM stage I or II. All patients underwent chemotherapy or surgical treatment at the FHSMU. This research was approved by the institutional review boards of the FHSMU, Shanxi Province.

#### 2.1.2. Determinant Variables

**Figure 2:**
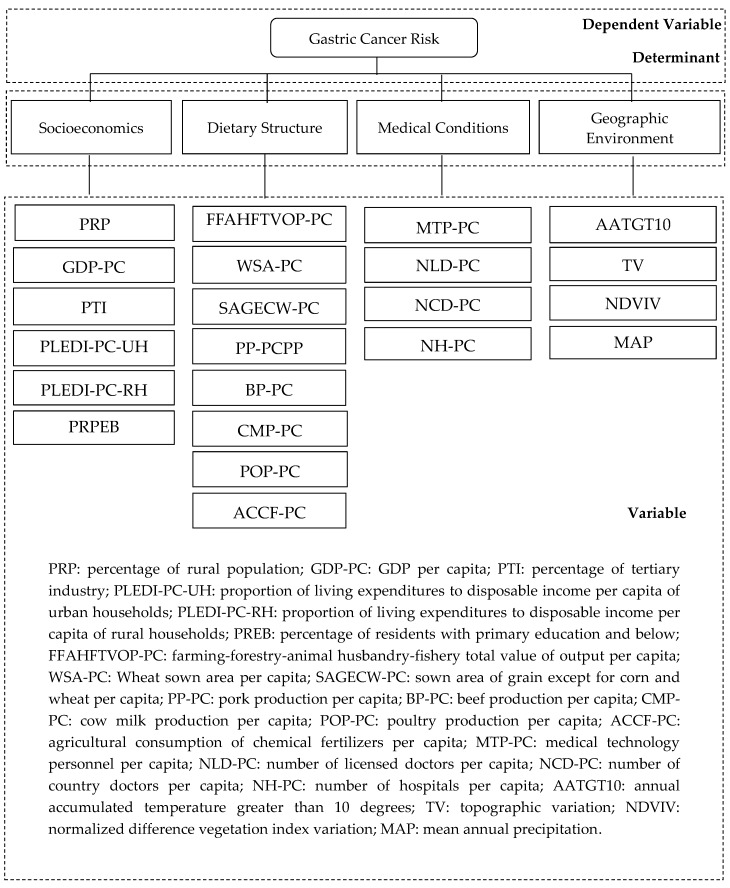
Gastric cancer risk determinants and their variables.

Based on previous research reviews, this paper investigated four types of GC risk factors: socio-economic, dietary structure, medical condition, and geographic environment, which included most non-genetic risk factors. Since cases were collected between 2014 and 2016, the year 2015 was defined as the baseline timepoint. The four categories of GC risk factors include 22 specific variables (Figure 2). The socio-economic influencing factor is represented by six variables: percentage of rural population (PRP), GDP per capita (GDP-PC), percentage of tertiary industry (PTI), proportion of living expenditures to disposable income per capita of urban households (PLEDI-PC-UH), proportion of living expenditures to disposable income per capita of rural households (PLEDI-PC-RH), and percentage of residents with primary education and below (PRPEB). The dietary structure influencing factor is represented by eight variables: farming-forestry-animal husbandry-fishery total value of output per capita (FFAHFTVOP-PC), wheat sown area per capita (WSA-PC), sown area of grain except for corn and wheat per capita (SAGECW-PC), pork production per capita (PP-PC), beef production per capita (BP-PC), cow milk production per capita (CMP-PC), poultry production per capita (POP-PC), and agricultural consumption of chemical fertilizers per capita (ACCF-PC). The medical condition influencing factor is represented by four variables: medical technology personnel per capita (MTP-PC), number of licensed doctors per capita (NLD-PC), number of country doctors per capita (NCD-PC), and number of hospitals per capita (NH-PC). The geographic environment influencing factor is represented by four variables: annual accumulated temperature greater than 10 degrees (AATGT10), topographic variation (TV), normalized difference vegetation index variation (NDVIV), and mean annual precipitation (MAP) from 1980–2015. These 22 variables were chosen based on the accessibility of these data. The first three influencing factor categories, which include 18 variables, were collected from the Shanxi statistical yearbook of 2015. The geographic environment influencing factor, which includes four variables, was provided by the Data Center for Resources and Environmental Sciences, Chinese Academy of Sciences (RESDC) (http://www.resdc.cn).

### 2.2. Methodologies

#### 2.2.1. Bayesian Spatial Statistical Model Integrated with a Selection Probability Model

Because of the small sample size, the Bayesian statistical method was used in this paper. The Bayesian spatial statistical model [45,46] has been widely applied in explorations of spatial trends of disease. Nevertheless, the case data in this paper were collected from a single hospital, the FHSMU; therefore, the Bayesian spatial model could not be directly applied. To correct bias, we presented a selection probability model. The main idea of the selection probability model is that the process of selecting hospitals for patients can be regarded as a stochastic process. If the selection probability of selecting the FHSMU for patients in various regions can be determined, then the actual patient number of the corresponding region may be estimated. 

For each patient, there are three options when selecting clinic hospitals: local hospitals, hospitals in provincial capital city, and hospitals in neighbouring provincial cities. The developed level is the primary factor of consideration when patients select clinic hospitals in local cities or outside of cities. In this paper, the developed level is represented by the developed grade of the city. Under the condition that patients have selected hospitals outside cities, the probability of selecting Taiyuan, the provincial capital city of Shanxi Province, is determined by the developed level and traffic distance. Figure 1a shows that there are four neighbouring provinces: Inner Mongolia, Shaanxi, Henan, and Hebei. Because of the traffic inconvenience (Figure 1b) and under-development of Inner Mongolia, patients in Shanxi rarely select hospitals in Inner Mongolia. Notably, although Beijing is not neighboured by Shanxi Province, Beijing’s hospitals have a strong attraction for Shanxi patients due to the high medical level in Beijing (Chinese capital city) and traffic convenience with Beijing (Figure 1b). Therefore, probable outside cities selected by Shanxi’s patients include four cities, Beijing, Xi’an, Shijiazhuang, and Zhengzhou (Figure 1). Taken together, the selection model can be expressed as follows:

(1) $$p_{i}=\frac{G_{TY\;}+G_{i\to }}{G_{i}+G_{TY}+G_{i\to }}\cdot \left(\frac{G_{TY}}{G_{TY}+G_{i\to }}\cdot \frac{\frac{1}{d_{i\to TY}^{2}}}{\frac{1}{d_{i\to TY}^{2}}+\frac{1}{d_{i\to }^{2}}}\right)\cdot \frac{1}{h}$$

where $p_{i}$ represents the probability of selecting the FHSMU for each GC case in the *i*-th city, which can be mainly disassembled to three portions: the probability of selecting outside cities, the probability of selecting Taiyuan city, and the probability of selecting the FHSMU in Taiyuan. The first two selection probabilities can be determined by the gravity model [47] and the inverse power-law traffic distance function by referencing a model of individual mobility [48]. In equation (1), $G_{i}$, $G_{i\to }$, and $G_{TY}$ represent the developed grade of the *i*-th city, Beijing or the provincial city of neighbouring province with the *i*-th city, and Taiyuan city, respectively. $d_{i\to TY}$ is the traffic distance from the *i*-th city to Taiyuan city. $d_{i\to }$ is the traffic distance from the *i*-th city to Beijing or the provincial city of neighbouring provinces. The coefficient h (h=5) is the number of the hospitals at the same level with the FHSMU in Taiyuan; we supposed that the GC patients’ selection probability of the top five hospitals with the same level in Taiyuan is equal. Additional, the random selection process can be regarded as a repeated Bernoulli process. Thus, the Bayesian spatial model may be expressed as follows:

(2) $$y_{i}\sim Bin\left(p_{i},C_{i}\;\right)$$

(3) $$C_{i}\sim Poisson\left(N_{i}*r_{i}\;\right)$$

(4) $$log(r_{i})=\alpha +S_{i}+\delta_{i}+\varepsilon$$

where $y_{i}$ is the number of GC cases in the *i*-th city of Shanxi collected from the FHSMU, $C_{i}$ is the number of GC cases in the *i*-th city by correcting bias. $N_{i}$ and $r_{i}$ are the population and GC morbidity of the *i*-th city in Shanxi, respectively. In formula (3), α represents the average level of GC risk throughout Shanxi, and is assigned to flat prior. $S_{i}$ represents the overall spatial component effects, and $\exp\left(S_{i}\right)$ directly quantifies the relative risk of the *i*-th city compared to Shanxi’s overall risk level, exp(α) [49]. The BYM model, named after its authors, Besag et al. [49], is a convolution of spatially structured and unstructured random effects, which is assigned to the parameter $S_{i}$. BYM considers both spatially structured random effects with a convolution algorithm and unstructured random effects using a normal distribution. The spatial structure effects are modelled using conditional autoregressive (CAR) [50]. The spatial adjacency matrix adopts the first order “Queen” form. The concrete form is as follows:

(5) $$S_{i}|S_{\left(-i\;\right)}\sim \mathrm{Normal}\left(\mu_{i}+\overset{n}{\underset{j=1}{\sum }}w_{ij}\left(S_{j}-\mu_{j}\right),\;\sigma_{i}^{2}\right)$$

where $S_{\left(-i\right)}=\left(S_{\left(j\right)}:j\neq i\right)$, $E\left(S_{i}\right)=\mu_{i}$, $E\left(S_{j}\right)=\mu_{j}$, $w_{ij}$ is the element of spatial adjacency matrix W, and $\sigma_{i}^{2}$ is the variance of $S_{i}$. $\delta_{i}$ represents spatial random effects. ε indicates a Gaussian noise error. Gaussian prior is assigned to $\delta_{i}$ and ε.

#### 2.2.2. Bayesian Lasso Regression Model

Considering the small sample size along with the 22 factors, this paper adopted the Bayesian Lasso regression model [51,52], which can overcome the problem of small sample size to some extent. The Bayesian Lasso regression model was developed from the Lasso regression, which differs from the ordinary least square (OLS), which is penalized by least squares that minimizes the residual sum of squares while controlling the L1-norm of the coefficient vector of regression:

(6) y=βX+ϵ

(7) $${\hat{\beta }}_{L}=argmin_{\beta }{\left(y-\beta X\;\right)}^{T}\left(y-\beta X\right)+\lambda {∥\beta ∥}_{1}$$

where λ≥0 determines the amount of shrinkage. In the view of Bayesian statistics, the Lasso regression can be interpreted as posterior mode estimates when the regression parameters have independent and identical Laplace priors [53]. The Bayesian lasso regression parameters were assigned by a prior conditional Laplace:

(8) $$\beta |\lambda ,\sigma \sim \overset{k}{\underset{i=1\;}{\prod }}\frac{\lambda }{2\sigma }e^{-\frac{\lambda \left|\beta_{i}\right|}{\sigma }}$$

where $\sigma^{2}$ is the variance of the conditional Laplace prior of the Lasso regression coefficient, β. k is the number of independent variables. The likelihood function of the observed data, y, fitted to a normal distribution:

(9) $$y|\beta ,\lambda ,\sigma \sim N\left(\beta X,\sigma^{2}I\;\right)$$

where I is an identity matrix. The meanings of the other parameters are the same as above. Then, the posterior of the regression parameter, β, can be expressed as follows:

(10) $$\beta |y,\lambda ,\sigma \sim \frac{1}{2\sigma^{2}\;}∥y-\beta X{∥}_{2}^{2}+\frac{\lambda }{\sigma }{∥\beta ∥}_{1}$$

According to Park and George’s study [52], we will regard $\lambda^{2}$ as the parameter rather than λ. This paper also considers the class of gamma priors on $\lambda^{2}$. The parameter $\sigma^{2}$ is assigned an inverse gamma prior. 

To investigate the factors in GC relative risk, the following formula was employed:

(11) $$\exp\left(S_{i}\;\right)=\beta_{1}X_{i1}+\cdots +\beta_{n}X_{in}+\epsilon$$

where $\exp\left(S_{i}\right)$ is estimated by the abovementioned Bayesian spatial statistical model. $\beta_{n}\;\left(n=1,\ldots ,n\right)$ is the regression coefficient responding to the n-th factor $X_{in}$. ϵ represents the Gauss random effect. Additionally, to remove the dimensional effect, all the variables were normalized by dividing their values by the corresponding provincial average value. 

The Bayesian statistics estimation in this paper was based on the Markov chain Monte Carlo (MCMC) algorithm. The Bayesian estimate of spatial variability was implemented in WinBUGS software [54], and the Bayesian Lasso regression used Pymc3 [55]. Two MCMC chains were run with different initial values. The number of iterations for each chain was set to 200,000; 150,000 iterations were for the burn-in period, and 50,000 were for the posterior distribution of parameters. Two MCMC chains were used to ensure the results’ convergence, which was evaluated by the Gelman-Rubin statistic [56]; the convergence is better when the Gelman-Rubin statistic is closer to one. The Gelman-Rubin statistics of each parameter in the paper were all between 0.9999 and 1.0001; the estimated results are thus reliable.

## 3. Results

### 3.1. Spatial Trends

**Figure 3:**
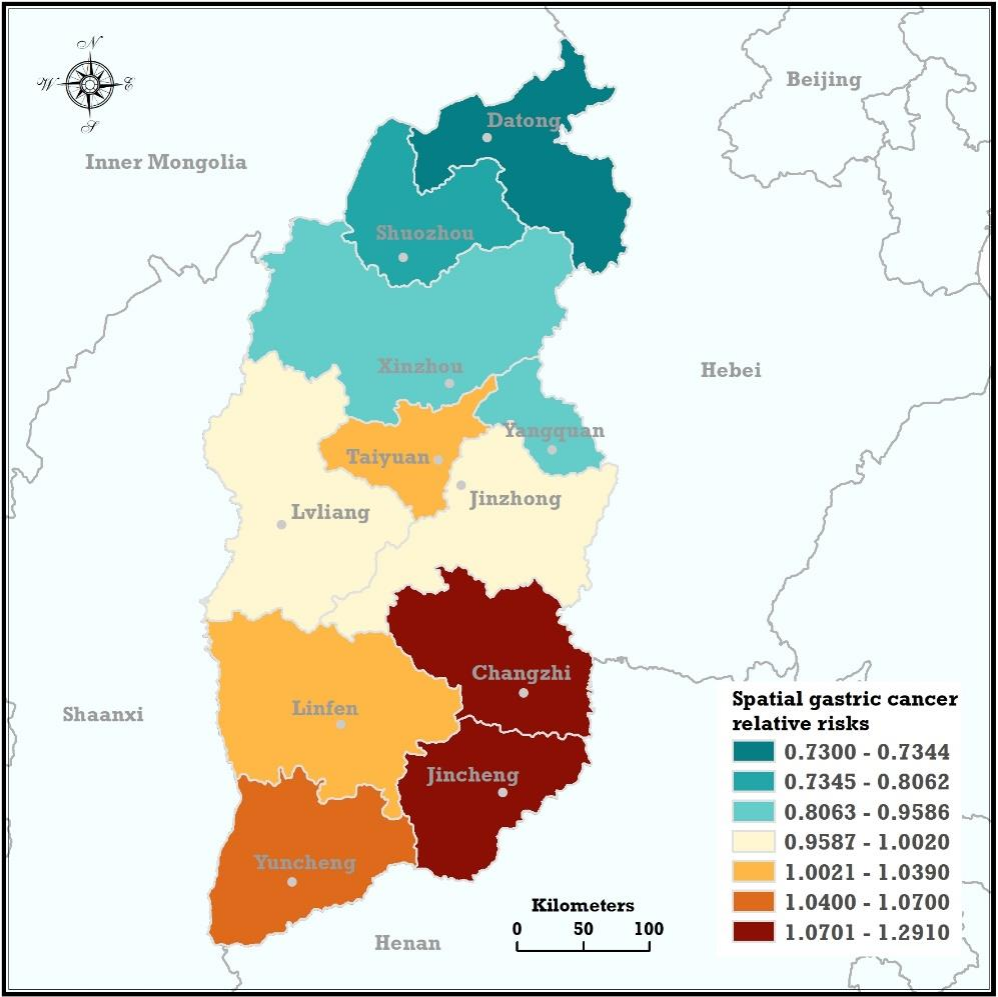
The spatial trends of GC relative risks based on the posterior medians of $\exp\left(S_{i}\right)$, estimated by the Bayesian spatial model integrated with the selection probability model.

The spatial GC relative risks can be quantitatively described by the posterior median of $\exp\left(S_{i}\right)$, whose value measures the relative magnitude of the GC incidence in the *i*-th city of Shanxi relative to the total provincial average incidence, exp(α). If $\exp\left(S_{i}\right)$ > 1.0, the GC incidence in the *i*-th city is $\exp\left(S_{i}\right)$ times the provincial overall GC incidence, and vice versa. Figure 3 shows the Shanxi spatial GC relative risks estimated from the Bayesian spatial statistical model integrated with the selection probability model based on the collected cases.

The estimated results showed that the spatial distribution of GC relative risks showed a distinct feature of being ‘high in the south and low in the north’. Two specific regions located in the southeast of Shanxi namely the south regions of Taihang Mountain, Jincheng and Changzhi had the highest GC spatial relative risk, with posterior medians of $\exp\left(S_{i}\right)$ of 1.291 (95% highest posterior density (95% HPD): 0.789–4.002) and 1.248 (95% HPD: 0.789–3.251), respectively. In addition, the top two high risk regions’ posterior probability of $\exp\left(S_{i}\right)$ > 1.0, which were denoted as $P(\exp\left(S_{i}\right)>1.0|\mathrm{Data})$, were 0.85 and 0.83, respectively. Yuncheng, Taiyuan, and Linfen also showed a higher spatial relative risk, with corresponding $P(\exp\left(S_{i}\right)>1.0|\mathrm{Data})$ values of 0.60, 0.63, and 0.60, respectively, and posterior medians of $\exp\left(S_{i}\right)$ of 1.070 (95% HPD: 0.514–2.257), 1.039 (95% HPD: 0.652–2.048), and 1.038 (95% HPD: 0.607–1.744), respectively. Lvliang and Jinzhong showed the provincial average GC risk. Their posterior medians of $\exp\left(S_{i}\right)$ were 1.002 (95% HPD: 0.5947–1.688) and 0.9913 (95% HPD: 0.556–1.571), respectively. Yangquan and the northern three cities, Datong, Shuozhou, and Xinzhou, had lower GC spatial relative risks than the overall provincial average. The population of the four cities with lower GC incidence accounted for 26.5% of the Shanxi’s population. 

### 3.2. Verification of Spatial Trends

The spatial trends of GC risk in Shanxi were estimated from the Bayesian statistical model with the selection probability model based on hospital-diagnosed case data. Given that the result of spatial trends is incorrect, it is difficult to make any further analysis, e.g., to analyse influencing factors. Although the result cannot be strictly verified due to the unavailability of survey data over Shanxi Province in recent years, some previous studies using GC survey data can be evaluated. Han and Zhao [57] have investigated Shanxi’s spatial distribution of GC based on disease survey data of GC in the late 20th century across Shanxi Province. Han and Zhao’s study [57] showed that the GC risk decreased as latitude increased in space, i.e., ‘high in the south and low in the north’. The conclusion based on GC survey data across Shanxi is consistent with the result in our study. Meanwhile, Han and Zhao noted that regions with high GC risk were located in the south section of Taihang Mountain and the surrounding areas, particularly Changzhi and Jincheng city. Our study reached the same conclusion that Changzhi and Jincheng city have the highest GC risk in Shanxi. Furthermore, according to the official announce of cancer epidemic survey data of six sampling areas collected by the Shanxi Cancer institute in 2009–2012, Taihang Mountain and the surrounding areas have the highest GC risk in Shanxi (http://health.sina.com.cn/news/2013-02-28/105674176.shtml). In addition, Wen et al. [58], Liang et al. [59], and Wen et al. [60] have all concluded that the south section of Taihang Mountain and the surrounding areas including Changzhi and Jincheng of Shanxi have a higher GC risk. Since these previous studies were all based on epidemiological survey data, these conclusions can be regarded as validation criteria. In sum, our estimated results of the spatial trends of GC risk over Shanxi coincide with the results based on GC epidemiological survey data, thus demonstrating the reliability of the method used in this paper. 

### 3.3. Influencing Factors

#### 3.3.1. Univariable Analysis

The GC relative risk of 11 cities in Shanxi Province estimated from the Bayesian spatial model integrated with the selection probability model was regarded as the dependent variable. The 22 influencing factors (Figure 2) of the 11 cities were regarded as the independent variables. Therefore, the associations between the GC relative risks and the 22 influencing factors were evaluated using Pearson correlation analyses. The statistical analysis showed that the *p*-value of the 10 factors was less than 0.10 (Table 1), whereas the other 12 factors were not significantly (*p* > 0.10) associated with the GC relative risk. The Pearson correlation coefficients (PCCs) for the relationships between the GC relative risk and the 10 factors were all greater than 0.40, and the corresponding statistical test *p* values were less than 0.10. 

There were five factors that significantly positively correlated with the GC risk, including the proportion of living expenditures to disposable income per capita of urban households (PCC = 0.65, *p* = 0.02), pork production per capita (PCC = 0.46, *p* = 0.08), annual accumulated temperature greater than 10 degrees (PCC = 0.62, *p* = 0.02), topographic variation (PCC = 0.59, *p* = 0.03), and mean annual precipitation from 1980–2015 (PCC = 0.83, *p* < 0.01); and 5 factors that negatively correlated with the GC risk, including the percentage of tertiary industry (PCC = −0.41, *p* = 0.10), sown area of grain except for corn and wheat per capita (PCC = −0.53, *p* = 0.05), beef production per capita (PCC = −0.60, *p* = 0.03), cow milk production per capita (PCC = −0.57, *p* = 0.03), and NDVI variation (PCC = −0.64, *p* = 0.02).

In high risk regions, dietary, agricultural and geographic environment factors had a more evident influence. In addition, the three dietary or agricultural factors, sown area of grain except for corn and wheat per capita, beef production per capita, and cow milk production per capita, were all associated negatively with GC risk. Amongst the four geographic factors, annual accumulated temperature greater than 10 degrees, topographic variation, NDVI variation, and mean annual precipitation from 1980–2015, only NDVI variation negatively correlated with the GC risk; the other 3 factors positively associated with GC risk.

#### 3.3.2. Multivariable Regression Results

The univariable analysis results cannot describe the synthesis and interaction effects of multiple factors that create multicollinearity, which can be observed from the PCCs between various variables (Table 1). To remove this multicollinearity effect, the Bayesian Lasso regression model was employed to investigate the combined associating effect of the 10 significantly influencing factors. Table 2 lists the estimated results, including the posterior mean of the regression coefficients inferred from the Bayesian Lasso regression model, the corresponding 95% HPD, and the posterior probability of the regression coefficients, $\beta_{n}$, greater than 0 or less than 0. According to the Bayesian hypothesis test theory [61], one way to decide between $H_{0}$ and $H_{1}$ is to compare $P\left(H_{0}|y\right)$ and $P\left(H_{1}|y\right)$ and accept the hypothesis with the higher posterior probability. This is the idea behind the maximum a posteriori test. 

The regression coefficient of the proportion of living expenditures to disposable income per capita of urban households, a social-economic factor, was the largest, at 1.20 (95% HPD: −1.06, 4.31, P($\beta_{4}$>0|y) = 82%). In addition, the other economic factor, percentage of tertiary industry, was the sixth-strongest influencing factor with a regression coefficient of −0.57 (95% HPD: −3.09, 1.16, P($\beta_{3}$<0|y) = 71%). The four geographic factors, annual accumulated temperature greater than 10 degrees, topographic variation, NDVI variation, and mean annual precipitation from 1980–2015, showed a certain influencing magnitude, with corresponding regression coefficients of 0.43 (95% HPD: −1.40, 2.73, $P\left(\beta_{20}>0|y\right)=$64%), 0.58 (95% HPD: −1.27, 2.52, $P\left(\beta_{21}>0|y\right)=73\%$), −0.81 (95% HPD: −3.04, 1.04, $P\left(\beta_{22}<0|y\right)=78$%), and 0.46 (95% HPD: −1.56, 2.98, $P\left(\beta_{23}>0|y\right)=65\%$), respectively.

The four dietary and agricultural factors sown area of grain except for corn and wheat per capita, pork production per capita, beef production per capita, and cow milk production per capita, showed different degrees of influencing magnitude. Thereinto, sown area of grain except for corn and wheat per capita and cow milk production per capita were the top two negatively influencing factors with regression coefficients of −0.20 (95% HPD: −1.06, 0.60, $P\left(\beta_{9}<0|y\right)=68\%$) and −0.22 (95%HPD: −0.64, 0.14, $P\left(\beta_{12}<0|y\right)=87$%), respectively. Pork production per capita and beef production per capita displayed a moderately positively and negatively influencing magnitude amongst the dietary and agricultural factors, with corresponding regression coefficients of 0.69 (95% HPD: −0.28, 1.68, $P\left(\beta_{10}>0|y\right)=$92%) and −0.68 (95% HPD: −1.78, 0.24, $P\left(\beta_{11}<0|y\right)=91\%$), respectively. Those two factors had an almost equal influence on GC.

## 4. Discussion

This paper explored the spatial variability of GC risk in Shanxi in north central China. To our knowledge, this is the first study to produce a GC disease map of a Chinese province at an urban scale in recent years. As mentioned before, disease mapping is generally produced based on survey data. We attempted to estimate the spatial trends of GC in Shanxi based on hospital-diagnosed case data, which must be corrected for bias. In this paper, a selection probability model was presented that aimed to correct this bias. Simultaneously, the Bayesian statistics paradigm was utilized to overcome the problem of small sample size. Although there are not direct survey data during the same period to verify our results, some previous studies [57,58,59,60] based on survey data pointed out the spatial distribution of GC or high risk regions of GC in Shanxi. Encouragingly, our estimated spatial trends of GC coincided with the previous research results, which demonstrates the reliability and feasibility of our methods. It is well known that obtaining disease survey data is difficult for a variety of reasons, including that performing disease surveys is a time and labour consuming work. Hence, we hope that this paper may contribute to mining not only GC hospital-diagnosed data, but also other cancers, e.g., lung cancer, oesophageal cancer, liver cancer, etc. 

The GC spatial trends can provide scientific evidence and references for relevant medical government departments to develop GC prevention policies. In clinical practice, most of the GC cases were diagnosed at late stages, when treatment is substantially less effective [62]. Hence, the accurate prevention or early diagnosis of GC is important in reducing GC incidence and mortality and to ease the GC disease burden. Based on the spatial distribution of GC risk, the relevant medical government departments may develop region-specific policies and utilize limited medical resources. The spatial trends of GC risk in Shanxi Province in north central China has the conspicuous feature of being ‘high in the south and low in the north’, which illustrates that GC risk is significantly different in various regions. This phenomenon indicates that GC incidence is related to regional factors, such as regional eating habits, local food structure [57], and geographic environment. This paper quantitatively assessed GC spatial relative risk compared to the provincial average risk level. However, future studies must be continuously conducted based on additional case samples. In addition, the spatio-temporal trends of GC risk should be investigated in future research. Based on the Bayesian estimated GC spatial relative risks, we evaluated influencing factors to GC using univariable analyses and a multivariable regression model that can synthetically assess the synthetical influencing magnitude of various factors. The estimated results show that all 10 influencing factors have the same positively or negatively associations resulted in the univariable analysis results (Table 1). Table 3 summarizes the correlations between GC risk and the four categories of factors, i.e., the 22 factors. Amongst the four types of factors, socio-economic, dietary structure, and geographic environment showed significant correlations with GC risk. However, medical condition factors were not significantly related with GC risk.

Socioeconomics is strongly associated with GC risk. Partially consistent with previous studies [40,41], we found evidence of associations between GC risk and several socio-economic factors. The regions where the percentage of tertiary industry was lower and PLEDI-PC-UH was greater had a higher GC spatial relative risk compared to the provincial average risk level. The factors percentage of tertiary industry and PLEDI-PC-UH belong to the socio-economic category; a higher percentage of the tertiary industry represents a more developed economic level, and vice versa. Meanwhile, a higher PLEDI-PC-UH implies lower savings, which could be considered a measure of a resident’s prosperity.

The statistical analysis showed that the regions with a lower percentage of tertiary industry and higher PLEDI-PC-UH, i.e., less developed economic level and less prosperity, had a higher GC risk. Nevertheless, the statistical analysis in this paper showed that other socio-economic factors, such as the percentage of rural population, GDP per capita, PLEIDI-PC-RH, and PRPEB, did not show significant associations with GC risk. When considering education level, previous studies show different results. Several previous studies [40,42,43] have reported that there is an inverse relationship between GC risk and the level of education. Gao et al. [34] found an opposite conclusion. In terms of regional epidemiology, we have not discovered a definite relationship between GC risk and education level. The results of the associations between GC risk and four dietary structure factors are a specific finding from this paper that can provide a feasible reference for governments when creating accurate regional guidelines for the prevention of GC. Specifically, sown area of grain except for corn and wheat per capita, beef production per capita, and cow milk production per capita associated negatively with the GC risk, whereas the pork production per capita is a positive influencing factor. Shanxi is known as the “Minor Coarse Cereal Kingdom” for its specific geographical position and climate features. In particular, the sowing area for minor coarse cereals in northern Shanxi, e.g., Datong, Shuozhou, and Xinzhou, is larger than that of southern Shanxi, such as Yuncheng and Jincheng. The larger the sowing area of minor coarse cereals, the greater the sown area of grain except for corn and wheat per capita. The residents of the regions where minor coarse cereals are sown in larger areas, namely, northern regions of Shanxi, have a diverse dietary structure. The residents living in the southern regions of Shanxi, Yuncheng, Linfen, Jincheng, and Changzhi, which are major wheat sowing areas, have a relatively singular dietary structure. Considering the GC risk feature of ‘high in the south and low in the north’, we conjecture that diversity in dietary structure may reduce GC risk. Moreover, a review [63] assessed the nutritional attributes of minor coarse cereals and stated that the nutrition in minor coarse cereals is helpful in reducing several types of chronic diseases such as cancer, cardiovascular diseases, and various gastrointestinal disorders. This finding verifies the inference in this paper from another perspective. Furthermore, we found a negative association between GC risk and cow milk and beef production. The possible mechanism is that milk contains several components with anticancer potential. This was reported in some studies [64,65]. In addition, several previous studies [66,67,68,69] have found similar conclusions, namely, an increased risk of GC in populations who consume less milk, whereas Gao et al. [34] reported that milk intake increases the risk of GC. The association between GC risk and beef has not reached a consensus yet. Ward et al. [70] and Huang et al. [71] reported that increased beef consumption was associated with a high GC risk. However, Chen et al. [72] drew an opposite conclusion. Chen et al. conducted a case-control study on upper gastrointestinal cancer (including GC) based on Shanxi GC cases. They found that beef consumption can reduce GC risk, which is consistent with this paper. Consistent with a few previous studies [73,74], we found that pork production per capita was positively associated with GC risk. The influencing mechanism of GC is a synthetical and multi-dimensional process, and we argue that the influencing mechanism of GC exerts various features in different regions. An influencing mechanism of GC with regional characteristics is displayed in Shanxi in north central China. According to recent cancer survey results from 12 sampling areas of Shanxi in 2009–2012 (http://health.sina.com.cn/news/2013-02-28/105674176.shtml), GC risk is associated with dietary habit and nutrition intake deficiency. It is well known that the nutritive values of beef are higher than those of pork, which may explain the associations between GC risk and beef production per capita and pork production per capita in Shanxi. Although geographic environment is also a crucial influencing factor for GC [13], there is limited relevant research. This paper quantitatively demonstrated the associations between four geographic factors and GC risk. The results showed that all 4 geographic environment factors, temperature, terrain, vegetation cover, and precipitation, were with high probability (greater than 60%) related to GC risk. In north central China, i.e., Shanxi, the higher the total temperature, namely, the greater the annual accumulated temperature greater than 10 degrees and mean annual precipitation from 1980–2015, the higher the GC risk. This result is in accordance with Han and Zhao’s [57] research based on survey GC data in the late 20th century across Shanxi. Topographic variation indicates the variability in terrain, which in a probability of 73% associated positively with GC risk. NDVI variation indicates the diversity of vegetation cover, which in a probability of 78% correlated negatively with GC risk. We speculate that the variability in terrain, vegetation cover and mean annual precipitation from 1980–2015 may determine local climate, which influences the health of regional inhabitants. The understanding of these concrete mechanisms requires further study.

There are some limitations in our study. The patient sample size was not large enough. The results would be more precise if additional patient data were included. Because we were limited in data collection, we explored the spatial variability of GC risk by employing Bayesian statistical paradigm. Although 22 factors were explored in this paper, other factors, such as the regional consumption of salt and the regional production of vegetables and fruit, were not involved. This assessment is the objective of the next study.

## 5. Conclusions

First, this paper presented a selection probability model and integrated it into the Bayesian spatial statistical model. This method can implement disease mapping from hospital-diagnosed patients. Second, the spatial trends of GC risk in north central China, i.e., Shanxi, showed a ‘high in the south and low in the north’ pattern. Third, this study employed the Bayesian Lasso regression model to detect the combined effects of the 10 significant (*p* < 0.10) factors inferred from the univariable analysis, and any factors did not have to be removed. Fourth, this paper also highlighted dietary structure and geographic environment as significant (*p* ≤ 0.08) factors associated with GC risk based on univariable analysis, and Bayesian Lasso regression model showed similar correlations in high probability (greater than 0.60).

## Figures and Tables

**Table 1:** The Pearson correlation coefficients between each pair of the GC relative risk and the 10 significantly associated factors.

Variables	GC-Relative Risk	PTI	PLEDI-PC-UH	SAGECW-PC	PP-PC	BP-PC	CMP-PC	AATGT10	TV	NDVIV	MAP from 1980–2015
**GC-relative risk**	1.00	−0.41	0.65	−0.53	0.46	−0.60	−0.57	0.62	0.59	−0.64	0.83
**PTI**	−0.41	1.00	−0.26	−0.17	−0.45	−0.14	0.22	−0.44	−0.40	0.22	−0.35
**PLEDI-PC-UH**	0.65	−0.26	1.00	0.00	0.47	−0.11	−0.08	0.04	0.10	−0.13	0.33
**SAGECW-PC**	−0.53	−0.17	0.00	1.00	−0.11	0.70	0.57	−0.59	−0.43	0.60	−0.63
**PP-PC**	0.46	−0.45	0.47	−0.11	1.00	0.00	−0.18	0.30	0.41	−0.29	0.67
**BP-PC**	−0.60	−0.14	−0.11	0.70	0.00	1.00	0.55	−0.65	−0.47	0.44	−0.59
**CMP-PC**	−0.57	0.22	−0.08	0.57	−0.18	0.55	1.00	−0.44	−0.55	0.40	−0.51
**AATGT10**	0.59	−0.40	0.10	−0.43	0.41	−0.47	−0.55	0.86	1.00	−0.21	0.64
**TV**	−0.64	0.22	−0.13	0.60	−0.29	0.44	0.40	−0.36	−0.21	1.00	−0.77
**NDVIV**	0.83	−0.35	0.33	−0.63	0.67	−0.59	−0.51	0.68	0.64	−0.77	1.00
**MAP from 1980–2015**	1.00	−0.41	0.65	−0.53	0.46	−0.60	−0.57	0.62	0.59	−0.64	0.83

PTI: percentage of tertiary industry; PLEIDI-PC-UH: proportion of living expenditures to disposable income per capita of urban households; SAGECW-PC: sown area of grain except for corn and wheat per capita; PP-PC: pork production per capita; BP-PC: beef production per capita; CMP-PC: cow milk production per capita; POP-PC: poultry production per capita; ACCF-PC: agricultural consumption of chemical fertilizers per capita; AATGT10: annual accumulated temperature greater than 10 degrees; TV: topographic variation; NDVIV: normalized difference vegetation index variation; MAP: mean annual precipitation.

**Table 2:** The Bayesian Lasso regression results of the 10 significant influencing factors.

Variables	$\mathrm{Posterior}\;\mathrm{Mean}\;\mathrm{of}\;\beta_{n}\;$	95% HPD	$\mathrm{The}\;\mathrm{Posterior}\;\mathrm{Probability}\;\mathrm{of}\;\beta_{n}>0$ $\;\mathrm{or}\;\beta_{n}<0$
PTI ($\beta_{3}$)	−0.57	(−3.09, 1.16)	$P\left(\beta_{3}<0|y\right)=$ 71%
PLEDI-PC-UH ($\beta_{4}$)	1.20	(−1.06, 4.31)	$P\left(\beta_{4}>0|y\right)=\;$82%
SAGECW-PC ($\beta_{9}$)	−0.20	(−1.06, 0.60)	$P\left(\beta_{9}<0|y\right)=68\%$
PP-PC ($\beta_{10}$)	0.69	(−0.28, 1.68)	$P\left(\beta_{10}>0|y\right)=\;$92%
BP-PC ($\beta_{11}$)	−0.68	(−1.78, 0.24)	$P\left(\beta_{11}<0|y\right)=91\%$
CMP-PC ($\beta_{12}$)	−0.22	(−0.64, 0.14)	$P\left(\beta_{12}<0|y\right)=87$%
AATGT10 ($\beta_{20}$)	0.43	(−1.40, 2.73)	$P\left(\beta_{20}>0|y\right)=\;$64%
TV ($\beta_{21}$)	0.58	(−1.27, 2.52)	$P\left(\beta_{21}>0|y\right)=73\%$
NDVIV ($\beta_{22}$)	−0.81	(−3.04, 1.04)	$P\left(\beta_{22}<0|y\right)=78$%
MAP from 1980–2015 ($\beta_{23}$)	0.46	(−1.56, 2.98)	$P\left(\beta_{23}>0|y\right)=65\%$

PTI: percentage of tertiary industry; PLEDI-PC-UH: proportion of living expenditures to disposable income per capita of urban households; SAGECW-PC: sown area of grain except for corn and wheat per capita; PP-PC: pork production per capita; BP-PC: beef production per capita; CMP-PC: cow milk production per capita; AATGT10: annual accumulated temperature greater than 10 degrees; TV: topographic variation; NDVIV: normalized difference vegetation index (NDVI) variation; MAP: mean annual precipitation.

**Table 3:** Summary of the association of risk factors and GC.

Four Types of Factors	Factors	GC
Socioeconomics	Percentage of rural population (PRP)	o
	Gross domestic product (GDP) per capita (GDP-PC)	o
	Percentage of tertiary industry (PTI)	−
	Proportion of living expenditures to disposable income per capita of urban households (PLEDI-PC-UH)	+
	Proportion of living expenditures to disposable income per capita of rural households (PLEDI-PC-RH)	o
	Percentage of residents with primary education and below (PRPEB)	o
Dietary structure	Farming-forestry-animal husbandry-fishery total value of output per capita (FFAHFTVOP-PC)	o
	Wheat sown area per capita (WSA-PC)	o
	Sown area of grain except for corn and wheat per capita (SAGECW-PC)	−
	Pork production per capita (PP-PC)	+
	Beef production per capita (BP-PC)	−
	Cow milk production per capita (CMP-PC)	−
	Poultry production per capita (POP-PC)	o
	Agricultural consumption of chemical fertilizers per capita (ACCF-PC)	o
Medical condition	Medical technology personnel per capita (MTP-PC)	o
	Number of licensed doctors per capita (NLD-PC)	o
	Number of country doctors per capita (NCD-PC)	o
	Number of hospitals per capita (NH-PC)	o
Geographic environment	Annual accumulated temperature greater than 10 degrees (AATG10)	+
	Topographic variation (TV)	+
	Normalized difference vegetation index (NDVI) variation (NDVIV)	−
	Mean annual precipitation (MAP) from 1980-2015	+

+: significant (*p* < 0.10) positive association or positive association with high probability (greater than 60%); −: significant (*p* < 0.10) negative association or negative association with high probability (greater than 60%); o: non-significant association.
